# Annexin A2 Heterotetramer: Structure and Function

**DOI:** 10.3390/ijms14036259

**Published:** 2013-03-19

**Authors:** Alamelu Bharadwaj, Moamen Bydoun, Ryan Holloway, David Waisman

**Affiliations:** 1 Departments of Biochemistry & Molecular Biology, Dalhousie University, PO Box 15000, Halifax, Nova Scotia, B3H 4R2, Canada; E-Mail: al229243@dal.ca; 2 Departments of Pathology, Dalhousie University, PO Box 15000, Halifax, Nova Scotia, B3H 4R2, Canada; E-Mails: mm841710@dal.ca (M.B.); rholloway404@gmail.com (R.H.)

**Keywords:** annexin A2, S100A10, phospholipid-binding, plasmin, fibrinolysis, plasminogen receptor, redox regulation, cancer, anti-phospholipid syndrome

## Abstract

Annexin A2 is a pleiotropic calcium- and anionic phospholipid-binding protein that exists as a monomer and as a heterotetrameric complex with the plasminogen receptor protein, S100A10. Annexin A2 has been proposed to play a key role in many processes including exocytosis, endocytosis, membrane organization, ion channel conductance, and also to link F-actin cytoskeleton to the plasma membrane. Despite an impressive list of potential binding partners and regulatory activities, it was somewhat unexpected that the annexin A2-null mouse should show a relatively benign phenotype. Studies with the annexin A2-null mouse have suggested important functions for annexin A2 and the heterotetramer in fibrinolysis, in the regulation of the LDL receptor and in cellular redox regulation. However, the demonstration that depletion of annexin A2 causes the depletion of several other proteins including S100A10, fascin and affects the expression of at least sixty-one genes has confounded the reports of its function. In this review we will discuss the annexin A2 structure and function and its proposed physiological and pathological roles.

## 1. Introduction

The annexins are a family of proteins that bind anionic phospholipids in a calcium-dependent manner (reviewed in [1–6]). Annexins were first discovered in animal cells and were named for their ability to “annex” or aggregate membranes [7]. The annexins are expressed in vertebrates (ANXA), invertebrates (ANXB), fungi and protozoa (ANXC), plants (ANXD) and protists (ANXE). The basic structural domains of the annexins consist of a variable amino-terminal head domain and a homologous carboxyl domain. The amino-terminal domain contains sites for post-translational modification and protein-protein interaction and imparts each annexin with a unique function(s). The carboxyl core domain is typically divided into four homologous domains (eight for annexin A6) of about 70 amino acids (numbered as domain I–domain IV), and each of these domains consist of five α-helices (A–E). The AB and DE helices are connected by loops, referred to as the AB and DE loops. One or more of domains I–IV contains a region of homology, called the endonexin fold, which include the residues of the AB loop and several amino acids that flank the AB loop. The endonexin fold is considered to be the signature amino acid sequence for the annexins, houses the calcium-binding motif (KGXGT-38 residues—D/E) and is present in at least one of the four AB loops. Most eukaryotic species have between one and twenty annexin (*ANX*) genes. The defining property that distinguishes annexins from other calcium-binding proteins is their calcium-dependent binding to negatively charged cellular membranes. However, while some annexins bind membranes in a calcium-independent manner, other annexins can insert into membranes as monomers or hexamers [8]. The annexins have also been shown to participate in a variety of membrane related functions such as exocytosis, endocytosis, the regulation of ion transport across membranes, membrane reorganization, vesicular trafficking and redox regulation (reviewed in [2,3,9]).

One member of the vertebrate annexins, annexin A2 (ANXA2) was initially identified as a substrate for the tyrosine kinase v-Src, the gene product of Rous Sarcoma virus which promotes cellular transformation [10]. Annexin A2 is present in various cells such as endothelial cells, monocytes, macrophages and most cancer cells. Annexin A2 can exist as a monomer or as a heterotetrameric complex with S100A10 (p11). The annexin A2-S100A10 complex is referred to asAIIt. The binding of S100A10 to annexin A2 regulates a number of functions of annexin A2, and thus the biochemical properties of monomeric annexin A2 and the heterotetrameric annexin A2 are distinct [11]. For example, the binding of S100A10 to annexin A2 reduces the calcium dependency of membrane interaction of annexin A2 from millimolar to micromolar levels of intracellular calcium. In contrast, annexin A2 plays an obligatory role in the regulation of S100A10 by protecting S100A10 from rapid ubiquitin-mediated degradation. Thus, S100A10 cannot exist in the absence of annexin A2 and depletion of cellular annexin A2 results in the rapid disappearance of S100A10 [12–17]. Recently it has been shown that the protein, deleted in liver cancer (DLC1) competes for the annexin A2 binding site in the carboxyl-terminal region of S100A10. Since the binding of DLC1 with S100A10 does not protect S100A10 from ubiquitin mediated degradation, S100A10 within this complex is ubiquitinated and degraded. This observation presents a unique mechanism for the regulation of S100A10 and S100A10-dependent plasmin generation at the cell surface. As presented in the graphical abstract, under normal conditions S100A10 protein is maintained at a steady-state level by the antagonist actions of DLC1 and annexin A2 on S100A10 stability. However, during oncogenic transformation, DLC1 levels decrease while annexin A2 levels increase. The increased formation of annexin A2-S100A10 complex increases the stability of S100A10 on the cell surface resulting in increased S100A10 mediated plasmin generation.

Extracellularly, annexin A2 primarily functions as a cell surface receptor for S100A10.. The S100A10 subunit plays a key role in maintaining vascular patency by regulating the cell surface generation of the proteolytic enzyme plasmin. In this regard, S100A10-dependent plasmin generation functions to stimulate the breakdown of fibrin blood clots (fibrinolysis) (recently reviewed in [18]). In addition, the annexin A2-S100A10 complex plays an important role in oncogenesis, also by regulation of the proteolytic activity of plasmin (recently reviewed in [19]). This cell surface plasmin activity is utilized by cancer cells, and tumor associated macrophages and neutrophils to digest the extracellular matrix and other tissue barriers. In addition, annexin A2 also interacts with extracellular receptors such as Toll-Like receptor 4 (TLR4) and ligands such as gastrin. Thus, AIIt-dependent cell surface plasmin activity empowers cancer and cancer-associated cells with the properties of invasion and metastasis. For example, macrophages use S100A10-dependent plasmin generation to allow movement through tissue barriers to sites of inflammation [20] Extensive studies of the annexin A2-S100A10 complex have demonstrated that the carboxyl-terminal lysine of the S100A10 subunit functions to bind plasminogen and the plasminogen activator, tissue plasminogen activator (tPA) which results in a dramatic stimulation of tPA-dependent conversion of plasminogen to plasmin. Therefore the function of annexin A2 in cell surface plasmin regulation appears to be indirect, *i.e.*, annexin A2 functions to transport S100A10 to the cell surface and anchor S100A10 to the extracellular surface of the plasma membrane (reviewed in [19,21]). Additionally, cell surface annexins A2 has been suggested to function in signal transduction.

## 2. Structure of Annexin A2 and AIIt Heterotetramer

**Figure 1:**
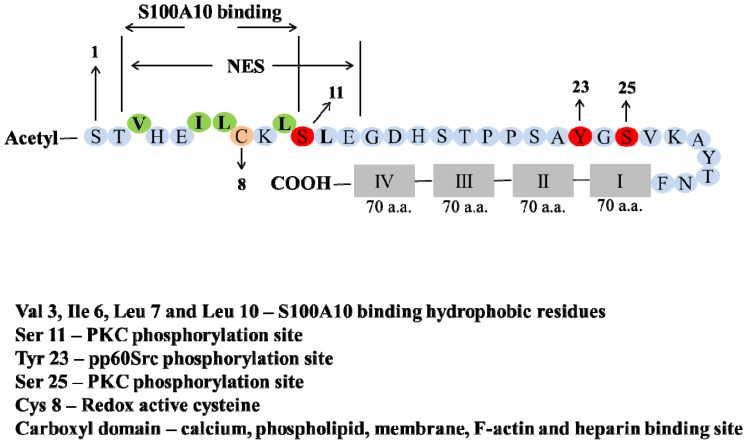
Domain structure of annexin A2. Annexin A2 is composed to two domains—the amino-terminal domain and carboxyl-terminal domain. The amino-terminal is the site for post-translational modifications (Ser-1–Phe-32) such as acetylation (Ser-1) and phosphorylation (Ser-11, Tyr-23, Ser-25). Additionally it also encompasses the redox reactive cysteine residue (Cys-8) and the nuclear export sequence (NES) (Val-3-Leu-12). The S100A10 binding site is an amphipathic α-helix, with the hydrophobic residues, Val-3, Ile-6, Leu-7 and Leu-10 making contacts with S100A10. The carboxyl-terminal core domain includes four predominantly alpha-helical domains each containing 70 amino acids. This carboxyl-terminal core domain contains binding sites for heparin and RNA, calcium and phospholipid and as well as for F-actin.

As described for the annexins in general, annexin A2 is composed of two major domains, the highly variant amino-terminal domain or head region and the conserved carboxyl-terminal core domain. The amino-terminal region is the site for post-translational modification, as well as ligand and protein interactions, while carboxyl-terminal core region contains binding site for calcium and anionic phospholipids, heparin, DNA and F-actin [22] (Figure 1). The carboxyl-terminal core domain consists of four repeat segments of 70 amino acids each called the annexin repeat. Each repeat segment contains five α-helices (A–E) with four of the helices oriented anti- parallel and the fifth one perpendicular to them [23] wound in a right-handed superhelix. The amino-terminal tail domain of annexin A2 contains a region of amphipathic alpha-helix, the hydrophobic surface of which binds to S100A10. Four hydrophobic amino acids of the amino terminus of annexin A2 (V3, I6, L7 and L10) form seven points of contact with helix HI of one S100A10 monomer, two points of contact with the hinge region of S100A10 and nine points of contact with helix HIV of the other S100A10 monomer, for a total of nineteen contact points between annexin A2 and S100A10 (reviewed in [19]). Moreover *N*-acetylation of annexin A2 was found to be necessary for forming the complex with S100A10. It was suggested that amino-terminal acetyl group stabilizes the helix dipole of annexin A2 amino-terminus required for formation of the region of amphipathic α-helix [24,25].

S100A10, the binding partner of annexin A2 belongs to a group of acidic, dimeric, small molecular weight, calcium-binding EF hand proteins. The calcium-binding sites in the two EF hand domains of S100A10 are unique among S100 proteins in that these domains are mutated such that they cannot bind calcium. Hence S100A10 does not undergo a calcium induced conformation change and assumes a conformation similar to the calcium bound states of other S100 proteins [26,27] (recently reviewed in [19]). The important functional domain of S100A10 is its carboxyl-terminal lysine residue which forms a binding site for tPA and plasminogen. Within the heterotetrameric complex S100A10 binds tPA and plasminogen with a Kd of 0.68 μM and 0.11 μM respectively.

Cryoelectron microscopy has suggested that each annexin A2 molecule binds to one membrane in the outer position of the heterotetramer, with S100A10 in the center of the complex [28]. Another study utilized scanning force electron microscopy to show that both annexin A2 molecules bind to the same membrane and the S100A10 dimer resides on the outer side of the heterotetramer thus facilitating interaction with other cytosolic proteins or receptors [29]. The former model possibly accounts for the membrane aggregation function of the AIIt heterotetramer. More recently, Schulz *et al.*[30] used chemical cross-linking, high resolution mass spectrometry and computational docking analysis to propose that the annexin A2/S100A10 complex exists on the inner cell surface as an octamer rather than as a heterotetramer as was originally proposed by our laboratory [11].

The amino-terminal domain of annexin A2, also possesses both serine and tyrosine phosphorylation sites [31] a reactive cysteine [32] and a nuclear export sequence [33]. Annexin A2 is planar and forms a slightly curved disc with a concave and convex side. Calcium, phospholipid binding and membrane attachment occur at the convex side, whereas the amino- and carboxyl-terminal S100A10 binding site occurs at the opposite concave side and faces the cytosol [3,24,25]. Using site-directed mutagenesis of annexin A2 and structures based on the crystal structure of annexin V, it has been suggested that type II calcium-binding sites are formed from a loop which connects the first and second alpha-helices (loop AB) of the second, third and fourth domain of the protein. An acidic residue, located about 38 residues downstream, between the fourth and fifth alpha-helices also comprises part of this calcium-binding site. Calcium-coordination is accomplished by 3 peptide carbonyl oxygen ligands from alternate loop residues in the AB loop sequence and by bidentate carboxylate oxygens from the distant acidic residue [34–38]. Annexin A2 also has two type III sites in the first repeat which are formed from three ligand-donating residues: two peptide carbonyl oxygens and a bidentate carboxylate group from a glutamic residue. Interestingly, only the type II calcium-binding sites play a role in membrane association of annexin A2. Moreover the association between annexin A2 and S100A10 which occurs on the concave side of annexin A2 does not require calcium [11]. Annexin A2 was initially crystallized by Luecke’s group. Unlike previous studies the annexin A2 analyzed by this group contained an intact amino-terminus. They reported that annexin A2 bound seven calcium molecules. Crystallographic analysis by Shao *et al.*[39] has demonstrated that annexin A2 shows significant calcium-dependent heparin binding both as a monomer and as the annexin A2-S100A10 complex. The heparin binding site was present at the convex face of the domain IV of annexin A2 and was formed by the two calcium-binding loops *i.e.*, IVAB and IVDE.

Classically annexin A2 has been described as a calcium-dependent phospholipid and membrane binding protein, where removal of calcium results in loss of phospholipid binding [3]. Interestingly, we reported that the mutagenesis and inactivation of the five calcium-binding sites (three type II and two type III) resulted in a protein that could bind to anionic phospholipid in the absence of calcium. Thus, the calcium-independent phospholipid binding reported in our mutational studies suggested that amino acids that form the calcium-binding sites might function to block access to phospholipid. These amino acids would allow access to phospholipid upon calcium-binding or due to their inactivation by mutagenesis [40].

## 3. Annexin A2 Binds to Anionic Phospholipids

Annexin A2 and the annexin A2-S100A10 heterotetramer have been shown to bind to and aggregate vesicles consisting of the anionic phospholipids, phosphatidic acid, phosphatidylserine and phosphatidylinositol but not vesicles composed of phosphatidylethanolamine or phosphatidylcholine [41]. When bound to a lipid bilayer, monomeric or heterotetrameric annexin A2 can form a monolayer of protein clusters with anionic phospholipids accumulating underneath these protein clusters [42]. Mounting evidence indicates that annexin A2 and AIIt are involved in organizing cholesterol-rich lipid rafts [43,44] and linking them to cytoskeletal proteins [45–48]. Although it was originally proposed that annexin A2 was a cholesterol binding protein it is now clear that annexin A2 does not bind cholesterol [49], but that cholesterol most likely enhances the membrane-binding affinity of annexin A2 by mediating the formation of anionic phospholipid-rich microdomains and/or clusters thus resulting in an increased local concentrations of anionic phospholipids. Since annexin A2 cannot effectively promote the formation of stable phosphatidylinositol 4,5-bisphosphate (PtdIns(4,5)P2) microdomains in the absence of cholesterol, cholesterol may play the dual role of promoting both the initial membrane adsorption of AIIt and the annexin A2-mediated formation of stable PtdIns(4,5)P2 microdomains. Monomeric and heterotetrameric annexin A2 is capable of binding to PtdIns(4,5)P2 with high specificity and affinity. This interaction also plays a role in the organization of actin at membrane sites that are enriched in PtdIns(4,5)P2 [6,50]. Together with previous reports showing that annexin A2 binds cholesterol-containing membranes [51,52] and PtdIns(4,5)P2 is localized in cholesterol-rich lipid rafts in the plasma membrane [53], these results identify a role for annexin A2 in the formation of PtdIns(4,5)P2-rich lipid raft-like structures. However, it is unclear if annexin A2 monomer or annexin A2-S100A10 heterotetramer is involved in these functions Nonetheless, Gokhale *et al.*[49] have shown that AIIt has at least 10 times higher affinity for PtdIns(4,5)P2-containing vesicles than the monomer indicating that intracellularly, AIIt and not annexin A2 monomer likely interacts with PtdIns(4,5)P2.

Gokhale *et al.*[49] also proposed a mechanism by which the annexin A2-S100A10 heterotetramer induced PtdIns(4,5)P2 clustering. They proposed that the annexin A2-S100A10 heterotetramer bound to anionic membranes via calcium-dependent electrostatic interactions. This was followed by lateral diffusion and targeting of the heterotetramer to PtdIns(4,5)P2 on the membrane surface. As the heterotetramer molecules laterally aggregated on the membrane surface, protein-bound PtdIns(4,5)P2 molecules also formed patches, a process which was facilitated by the membrane cholesterol. It was also suggested that the heterotetramer-induced PtdIns(4,5)P2 clusters would be accessible to other PtdIns(4,5)P2-binding proteins. This allows the formation of discrete regions of the membrane containing clusters of specialized proteins. Since the heterotetramer can also interact with the F-actin cytoskeleton, it was proposed that the movement of these PtdIns(4,5)P2 clusters would be severely restricted.

**Figure 2:**
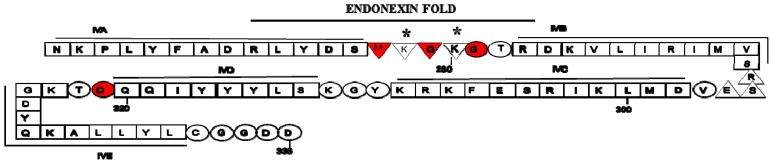
Phospholipid binding domain of annexin A2. For simplicity only the fourth domain (IV) of annexin A2, encompassing the five regions of alpha-helix (helix IVA to helix IVE) is presented. This cartoon illustrates the location of the endonexin fold (Arg-272-Arg-283). The calcium and phospholipid binding sites are located in the AB loop connecting helix IVA to helix IVB and also include the distal aspartic acid residue. The * indicates the lysine residues shown to participate in binding PtdIns(4,5)P2.

Seaton and coworkers initially characterized the interaction of annexin V with anionic phospholipid [54]. They demonstrated that the Gly and adjacent Thr in the AB loop (Gly-281 and Thr-282 in Figure 2) participate in calcium and phosphatidylserine binding. They observed that the carbonyl oxygen of the Gly residue coordinated binding of the AB calcium while its amide interacted with the glycerol backbone of the phospholipid. The Thr carbonyl oxygen coordinated a second (AB’) calcium while its methyl group served to stabilize the calcium-binding loop and its hydroxyl group formed a hydrogen bond with the amino group of the phosphatidylserine. They also identified highly specific interactions between the serine head group of phosphatidylserine and annexin V. For example, the phosphoserine oxygen coordinated the AB calcium while the serine carboxylate coordinated the AB’ calcium. Besides linking these two calcium ions, the alpha amino group of phosphatidyl serine was observed to hydrogen bond with the side-chain hydroxyl of the Thr residue and to also hydrogen bond to the carbonyl oxygen of the distal Glu residue that forms bidentate carboxylate oxygen ligands for the AB calcium. Interestingly, the region of annexin A2 that provides the binding site for PtdIns(4,5)P2-binding has been shown to involve Arg-273-Arg-284 [49]. This site corresponds to the basic nine-amino acid motif that has been identified in several F-actin- and PtdIns(4,5)P2-binding proteins [55]. These authors showed that Lys-278 and Lys-280, are directly involved in PtdIns(4,5)P2 binding. These residues are located in the domain IVAB calcium-binding loop and the carbonyl groups of the residues adjacent to the PtdIns(4,5)P2 binding residues (Met-277, Gly-279 and Gly-281) directly participate in calcium-binding (Figure 2).

## 4. Transcriptional Regulation of Annexin A2

Annexin A2 expression is regulated at both the transcriptional and translational levels. Specifically in cancer cells, it is transcriptionally regulated by growth factors such as insulin, fibroblast growth factor and epidermal growth factor [56]. It is also induced in cells transformed by v-*src*-, v-H-*ras*-, v-*mos*-, or SV40 [57]. For instance, c-Fos a major component of the transcription factor AP-1 induces annexin A2 expression in rat fibroblasts and neuronal PC12 cells [58]. It was demonstrated that annexin A2 expression was increased both at the transcript and protein level *in vitro* in chondrocytes and retinal endothelial cells and in a murine model of ischemic retinopathy. This was primarily through a Vascular Endothelial Growth Factor (VEGF)/VEGF-R2 and PKCβ pathway [59,60]. Annexin A2 is also up-regulated in osteoblastic cells in conditions of hypoxia, however via a VEGFR1/Neuropilin pathway and Src and MEK kinase pathway [59]. These pathways implicate the importance of transcriptional regulation in the modulation of annexin A2 levels.

Additionally annexin A2 is subject to regulation by alternative splicing in humans, mice and rats. Alternative splicing produces different isoforms of annexin A2 differing in the amino-terminal regions. Since the amino-terminal region is involved in various functions, alternative splicing has the potential of playing an important role in the function of annexin A2. Human annexin A2 mRNA is alternatively spliced into two isoforms, one which is the canonical annexin A2 consisting of 338 amino acids and the other is a minor form with additional 18 amino acids at the amino-terminal end. It is possible that this form does not bind to S100A10, since it lacks the serine residue that is acetylated in the major form of annexin A2 [3,61]. Similarly, alternative splicing of annexin A2 transcripts in mice results in the formation of two isoforms, one of which has low abundance. The splicing occurs in the 5′-untranslated region (5′-UTR), in which a 70 nucleotide non-coding exon2 insertion between the exon 1 and exon 3 results in difference in translation and transport of the mRNA between the two isoforms [62]. In rats isoform 2 an additional 6 nucleotides between exon 3 and 4 translates into and additional serine residue in the amino-terminal region, and provides another serine phosphorylation site [63]. Interestingly, annexin A2 also regulates its own expression by binding to its mRNA within the 3′-UTR region. The binding occurs within a region of 100 nucleotides with two repeats of the consensus sequence 5′-AA(C/G)(A/U)G in the mRNA [64] thus regulating its own mRNA transport and translation.

## 5. Post-Translation Modification and Regulation of Annexin A2

The amino-terminal region contains sites for post-translational modification and serves to regulate the properties of both amino and carboxyl-terminal domains [2]. Thus, the amino-terminal domain has sites for acetylation, serine and tyrosine phosphorylation, and glutathionylation. These post-translational events regulate the nuclear export sequence and S100A10 binding site that are housed in the amino-terminal domain. Acetylation of Ser-1 in the amino-terminal domain regulates the binding to S100A10 [61]. These modifications of amino-terminal region typically regulate the F-actin and phospholipid-binding activity of the carboxyl-terminal core domain [65].

Annexin A2 has been shown to be poly-ubiquitinated in porcine intestinal mucosa and mouse Krebs II cells. Interestingly this modification does not result in proteosomal degradation of annexin A2; rather it promotes its enrichment in the cytoskeletal fraction. This potentially has a role in F-actin and lipid raft binding due to the association of the ubiquitinated form with triton-insoluble fractions [66]. In a recent study by Deng *et al.*[67] the poly-ubiquitinated annexin A2 was shown to be elevated in breast cancer tissue although the functional significance of this observation is not established.

We have observed that loss of S100A10 also affects annexin A2 protein levels in a tissue-specific manner. Specifically, S100A10 null-mice show decreased levels of annexin A2 in the lungs, liver, spleen and kidneys, whereas the levels in the intestine were not affected. The mRNA levels in all the tissues remained unchanged. Thus, it appears that S100A10 can reciprocally regulate annexin A2 protein stability, although the mechanism of such a regulation is not understood [19].

Since the annexin A2-S100A10 complex plays an important role as a cell surface plasminogen receptor (discussed later), its secretion and localization to the extracellular surface is an essential regulatory step. The absence of a signal peptide in annexin A2, suggests that it could be secreted by one of the non-conventional secretion pathways [68–70]. There are three potential ways by which annexin A2 secretion to the extracellular surface could be accomplished. First, annexin A2 is incorporated into multivesicular endosomes and subsequently released as exosomes. Interaction with S100A10 and phosphorylation of Tyr-23 is essential for this process [71,72]. Secondly, SNARE-mediated fusion with plasma membrane could also result in the movement of the AIIt complex outside the cell as seen in enterocyte brush border cells [73]. Lastly, binding of annexin A2 to PtdIns(4,5)P2 could promote the formation of transmembrane channel or pore analogous to secretion of FGF2 [70]. All these three processes require the presence of S100A10, thus suggesting that it plays a key role in many of the functions proposed for annexin A2.

## 6. Phosphorylation

Phosphorylation is an important post-translational modification that controls the activity and functions of various cellular proteins such as enzymes, receptors, ion channels and regulatory or structural proteins. Several growth factors such as platelet derived growth factor receptor; hepatocyte growth factor and insulin growth factor induce tyrosine phosphorylation of annexin A2. Nicotine stimulation of adrenal cells causes PKC activation which induces serine (Ser-25) phosphorylation of annexin A2 and calcium- dependent exocytosis [74,75]. One of the other critical residues in annexin A2 that is phosphorylated in response to several stimuli described above is Tyr-23, located in the amino-terminal tail region of the protein. In the initial studies during identification of annexin A2, tyrosine phosphorylation has shown to occur both *in vivo* and *in vitro* by pp60vsrc [10,76,77] and was later shown to be at Tyr-23 [31]. The phosphorylation event reduces annexin A2 binding to phospholipid vesicles at low calcium concentration, and thereby prevents binding to F-actin and bundling *in vitro*[65] whereas Ser-25 phosphorylation of AIIt by PKC inhibits its ability to aggregate phospholipid vesicles [78,79] without affecting lipid vesicle binding of the protein. Moreover PKC phosphorylation of annexin A2 at Ser-11 reduced its association with S100A10, [25] and caused dissociation of the annexin A2-S100A10 complex [80,81]. Deora *et al.*[71] have shown that temperature stress induced translocation of annexin A2 to cell surface is dependent on its association with S100A10 as well as its tyrosine phosphorylation. Other studies have [82] has demonstrated that oncogenic v-src dependent annexin A2 phosphorylation plays an important role in inducing cell scattering and branching morphogenesis in MDCK cells by modulating cofilin-dependent actin dynamics [82]. This could implicate the involvement of annexin A2 in epithelial-mesenchymal transition (EMT) and tumor metastasis. Consistently a recent investigation by Lei Zheng *et al.*[83] has shown that Tyr-23 phosphorylation of annexin A2 is required for Rho-regulated EMT process in mouse model of pancreatic cancer. Ser-25 and Ser-11 phosphorylation of annexin A2 also prevents the nuclear shuttling of the protein in prostate cancer cells [84]. Paradoxically, Eberhard *et al.*[33] have shown that tyrosine phosphorylation of annexin A2 promotes the nuclear entry of the protein. They suggest that such a process requires annexin A2 to dissociate from S100A10. Moreover the Hajjar group has shown recently that plasmin induces PKC activation and Ser-25 and Ser-11 phosphorylation of the annexin A2, resulting in its dissociation. This was suggested to act as a feed-back loop preventing further activation of plasmin on the cell surface by S100A10 [13].

Insulin receptor tyrosine kinase also phosphorylates Tyr-23 of annexin A2 [85]. It was suggested that the phosphorylation of annexin A2 is an early event in insulin receptor activity and correlates with the initial steps in insulin receptor endocytosis and sorting. It was proposed that phosphorylation of annexin A2 resulted in its association with insulin receptor at early endosomes, which prevents fusion with lysosomes and further degradation of insulin receptor [86]. More recently it has been demonstrated that insulin induced Tyr-23 phosphorylation of annexin A2 in BHK cell line causes the remodeling of membrane associated-actin and cell detachment (loss of cell adhesion) by converting actin from a stationary to a more motile phenotype [87]. Collectively, reports from multiple laboratories suggests that phosphorylation of annexin A2 at different residues could have different effects on its localization and functional activation.

## 7. Redox Regulation

Cysteine residues in proteins are one of the common amino acids that undergo reversible oxidation/reduction under physiologic conditions [88]. The cysteine residue acts as a redox sensor that regulates protein structure and function. Reactive oxygen species (ROS) which encompasses hydrogen peroxide (H_2_O_2_), superoxide anion (^·^O_2_^−^), singlet oxygen (^1^O_2_) and hydroxyl radical (^·^OH) play an important role in various cellular processes such as proliferation, differentiation, autophagy, cell cycle arrest, senescence, apoptosis and necrosis [89–91]. Excessive ROS results in DNA damage, lipid peroxidation and irreversible protein damage and loss of function. The redox sensitive cysteines in proteins undergo various oxidative modifications by ROS such as formation of sulfenic acid, sulfinic acid, sulfonic acid, disulphide bonds and nitrosothiols derivatives [92]. Redox sensitive cysteines are those that exist in the Cys-*S*- form at neutral pH. In contrast, the Cys-SH form of cysteine is the non-reactive and common species present in proteins. Since the pKa value of protein cysteine residues is 8.5 the majority of cysteine residues exist as Cys-SH at physiological pH. ROS are rapidly eliminated by anti-oxidant enzyme systems such as catalase, superoxide dismutase, glutathione peroxidase, and peroxiredoxins. However various cellular stresses such as radiation, drugs, temperature and pH changes, nutrient deprivation can promote increased levels of ROS, causing cellular oxidative stress. In such situations, the oxidized cysteine in proteins acts as a sensor of change in redox potential and stimulates signaling pathways activating the cellular antioxidant responses.

Annexin A2 has four cysteine residues—Cys-8, Cys-132, Cys-261, and Cys-334. Among these residues, Cys-8 and Cys-334 are present on the amino- and carboxyl- terminus respectively. These residues are on the convex side of the protein facing the cytoplasm. Based on crystallographic studies, it has been suggested that Cys-132 and Cys-261 forms disulphide linkages in the annexin A2 monomer [93]. Cys-334 is only exposed at the surface when annexin A2 binds to plasmin, and is involved in cleaving disulphide bond in plasmin [94]. The function of Cys-8 is well characterized and suggested to be the redox sensitive cysteine. The first study to highlight this role of Cys-8 was in HeLa cells. Treatment with TNF-α in the presence of a glutathione analogue resulted in *S*-glutathionylation of Cys-8 of annexin A2 [95]. At that time the physiological relevance of this modification was not identified. Further studies showed that chemical modification of AIIt by the sulfhydryl reagent *N*-ethylmalemide or peroxnitrite results in loss of liposome aggregation of annexin A2 *in vitro*[96,97]. Our laboratory carried out a detailed investigation of reversible glutathionylation of annexin A2 and effect of modification on its function. In this study we showed that oxidative stress induced glutathionylation of Cys-8 and Cys-132 of the annexin A2 subunit of AIIt caused the loss of phospholipid and F-actin binding [98]. Interestingly, we showed that AIIt was also de-glutathionylated by glutaredoxin, which resulted in reactivation of its phospholipid and F-actin binding activity. In other studies, we showed that the thiols of AIIt participated in plasmin reduction promoting the release plasmin cleavage product, angiostatin. The reduction of plasmin at the cell surface in HT1080 fibrosarcoma cells by AIIt resulted in the oxidation of AIIt. The oxidized AIIt was further reduced by thioredoxin system. This study demonstrated the role of AIIt in redox-dependent processing of plasminogen and also identified AIIt as a substrate for the thioredoxin system [94].

Recently we have identified a novel redox regulatory role for annexin A2 critical for tumorigenesis [99]. We observed that annexin A2–null mice showed increased oxidation of liver and lung tissue proteins compared to WT mice. Furthermore, proteins isolated from tumor cells depleted of annexin A2 also show more oxidation compared to control cells. We showed that Cys-8 residue of annexin A2 is an important redox-sensitive cysteine which is reversibly oxidized by H_2_O_2_ and then reduced by the thioredoxin system. Thus, annexin A2 possibly acts an anti-oxidant in oxidative stress conditions within the cells by degrading H_2_O_2_. Consequently, we have observed that annexin A2 depleted tumor cells present with significantly increased levels of ROS, and also showed increased oxidation of redox sensitive cellular proteins upon H_2_O_2_-mediated oxidative stress. Furthermore tumors formed by subcuteaneous injection of annexin A2 depleted cells into immunocompromised mice showed impaired growth. Tumor growth of the annexin A2 depleted cells was rescued by application of the anti-oxidant *N*-acetyl cysteine (NAC). Taken together, these results delineate an important role of annexin A2 in tumor growth by functioning as a redox-regulatory protein. This study has significant implications in cancer chemotherapeutics and radiation therapies which largely function by increasing the ROS levels within a tumor cells. Annexin A2 possibly provides chemoresistance to tumor cells by acting as an anti-oxidant and protects cellular components against oxidative damage. This is consistent with reported up-regulation of annexin A2 in chemoresistant breast tumor cells [100].

A small percentage of total cellular annexin A2 has been shown to be present in the nucleus, though predominantly it is found in the cytoplasm and plasma membrane [33,101,102]. The nuclear export sequence (NES) in annexin A2 located in the amino-terminal tail region prevents its accumulation in the nucleus. This sequence consists of T-2VHEILCKLSL-12 and is similar to other reported NES *L*-(X1-4)-*L*-(X2)-*L*-(X)-*L*, where *L* is usually a hydrophobic residue. The NES also overlaps the S100A10 binding sequence (VHEILCKL). Since it is unlikely that the hydrophobic residues that participate in S100A10 binding could also participate in nuclear exclusion it was anticipated that the annexin A2-S100A10 complex would accumulate in the nucleus and not be exported. However, we have observed that although annexin A2 is easily detected in the nucleus, S100A10 does not appear to be present. It is unclear how annexin A2 is transported into the nucleus as the protein does not possess a recognizable nuclear import sequence. It is reasonable to propose that the binding of S100A10 prevents the transport of annexin A2 into the nucleus.

The function of nuclear annexin A2 is not clearly understood though it has been shown to bind RNA [103] and has also been suggested to participate in primer recognition protein complex that is involved in enhancing DNA polymerase α activity [104]. We have recently shown that the nuclear accumulation of the annexin A2 monomer plays a role in protecting the cells from DNA damage during oxidative stress [105]. Rapid nuclear accumulation of annexin A2 occurred in response to DNA damaging agents such as gamma radiation, UV radiation, chromium VI, and etoposide and under conditions of H_2_O_2_- mediated oxidative stress. Importantly the nuclear accumulation was mediated by inactivation of the NES and was independent of oxidation of the Cys-8 residue in annexin A2. This suggests that the annexin A2 which accumulates in the nucleus has a reactive thiol (Cys-8) which is capable of reacting with ROS such as H_2_O_2_. Moreover the annexin A2 depleted cells are more sensitive DNA damage as seen by the formation of phosphorylated H2AX-53BP1 foci in the nucleus. Consistent with our study, Weber’s group revealed that annexin A2 translocates to the nucleus in human organotypic culture and murine epidermal cells on exposure to X-radiation. In this investigation, annexin A2 depleted cells show increased sensitivity to TNF-α induced apoptosis, [106]. The redox function of annexin A2 may be relevant in designing novel can cancer therapeutics that are aimed at increasing the effectiveness of radiation and chemotherapeutics which largely function by increasing ROS levels.

## 8. Functions of Annexin A2

### 8.1. F-Actin Binding

Complexes of the annexin A2 monomer and heterotetramer and actin was first demonstrated in proteins purified from brush borders of intestinal epithelial cells [107], and in A431 cells and fibroblasts [108]. Subsequent studies by our laboratory showed that AIIt is a high affinity F-actin binding protein [109]. This high affinity binding occurred in the presence of calcium and in a cooperative manner. At physiological levels of calcium, F-actin binding to AIIt resulted in F-actin bundling. Although bundling was observed for the monomer, it was to a lesser extent and required a significantly higher concentration compared to AIIt [109]. A synthetic nonapeptide which corresponds to residues 286–294 of annexin A2 resulted in the loss of the calcium-dependent F-actin bundling, but not binding [110]. This suggested that binding of F-actin to AIIt results in a conformation change which enhances AIIt’s ability to interact with other AIIt molecules leading to bundling. The F-actin bundling activity of AIIt is decreased by Tyr-23 phosphorylation of AIIt [65]. Further to this, our laboratory also mapped the F-actin binding region of AIIt to the carboxyl-terminal amino acid residues LLYLCGGDD [111]. The F-actin bundling activity of AIIt is decreased by Tyr-23 phosphorylation of AIIt [65]. More recently it has been shown that the monomer is able to bundle pre-formed F-actin filaments and is also capable of regulating the growth of newly formed filaments [112]. Interestingly, although AIIt is an actin bundling protein it does not play a role in stress fiber or filopodia formation. It is rather associated with dynamic membrane cytoskeletal structures such as rocketing macropinosomes [47] and phagosomes [113]. Thus, depletion of annexin A2 results in the enrichment of stress fibers and loss of dynamic ruffling physiology [112].

Formation of actin networks is regulated by proteins such as Arp2/3 [114], WASP, WAVE and SCAR. These proteins are in turn regulated by small GTPases such as Rho, Rac1 and Cdc42. Annexin A2 was purified from complexes containing Rac1 at actin rich cell-cell contacts [115]. Additionally, annexin A2 associates with AHNAK at the cytosolic surface of the plasma membrane of cell-cell contacts in MDCK cells [45]. In another example annexin A2 was associated in submembranous actin during clustering of the hyaluronan receptor CD44 [44]. Actin-dependent transport of secretory vesicles to the apical membrane also required the presence of annexin A2 in epithelial cells [116]. In summary, annexin A2 acts as a scaffold protein which links actin cytoskeleton and various membrane microdomains or recruits factors for actin remodeling events.

### 8.2. Exocytosis and Endocytosis

Very early studies using electron microscopic analysis had shown that annexin A2 forms cross-links between secretory granules and the plasma membrane in stimulated neuroendocrine cells. Nonetheless, the involvement of annexin A2 in exocytosis remained controversial. For example introducing an inhibitory peptide competing for interaction of annexin A2 with S100A10 in chromaffin cells had no effect on secretion [117,118]. Moreover, expression of a chimeric protein that causes aggregation of annexin A2 in the cytosol did not affect secretion in PC12- adrenal medulla cells [119]. In contrast, a synthetic peptide corresponding to the amino-terminal region harboring the PKC phosphorylation site in annexin A2 was capable of inhibiting catecholamine secretion in chromaffin cells [120]. Moreover PKC dependent phosphorylation of annexin A2 was required for generating a secretory response by stimulated chromaffin cells [75]. These results suggest the requirement for the PKC phosphorylation in annexin A2-mediated secretion. AIIt may play an important role in exocytosis not only by forming and stabilizing lipid microdomains in the plasma membrane, but also by organizing the exocytotic machinery in the chromaffin cells [121]. Through its ability to bind actin, AIIt also participates in the formation membrane cytoskeletal complexes which control lipid raft assembly and the formation of functional exocytotic sites.

As described earlier, plasma membrane-associated annexin A2 binds PtdIns(4,5) P2-containing vesicles. This is important since PtdIns(4,5)P2 forms lipid microdomains in the plasma membrane in close proximity to SNARE complexes during exocytosis and endocytosis [122]. In fact, annexin A2 is targeted to the endosomal membrane/compartment, through a specific targeting sequence in the amino-terminal interaction domain [123]. Interestingly, this endosomal association occurs at membrane sites characterized by high cholesterol content [46,52]. Some reports have suggested that annexin A2 physically interacts with cholesterol forming cholesterol rich platforms on early endosomes, which also involves interactions with other proteins. This property of annexin A2 is independent of the presence of S100A10, suggesting that S100A10 is not essential for endosome binding.

Cruetz *et al.*[124] have shown that annexin A2 also plays a role in clathrin mediated endocytosis. Annexin A2 interacts with the μ2 subunit of clathrin assembly proteins AP2 via the *YXX*ϕ (*Y*—Tyr 23, *X*—variable residue, ϕ—bulky hydrophobic residue) sequence present in the amino-terminal region of annexin A2. Interestingly, PtdIns(4,5) P2 which bind to annexin A2, also regulate the binding of *YXX*ϕ motifs to μ2. A recent investigation of APPL endosomes revealed that annexin A2 interacts with the two Rab5 effector proteins APPL1 and APPL2 [125]. Rab5 a GTPAse is a dominant regulator of early steps of endocytosis [126]. As a component of APPL endosomes, annexin A2 is required for endosomal localization of APPL2 along with Rab5, since loss of annexin A2 results in loss of APPL proteins and APPL endosomes [125]. Unfortunately whether annexin A2 links actin to APPL proteins could not be established by these studies since loss of annexin A2 leads to direct disruption of APPL endosomes.

Another aspect of annexin A2 involvement in endocytosis is mediated by its property of binding to actin in a calcium dependent manner. Annexin A2 could potentially serve as a link between actin cytoskeleton and clathrin-coated vesicles. Moreover annexin A2 can also link different domains of early endosomes via calcium independent cholesterol binding as described previously. However, Valapala and Viswanatha have described the calcium-dependent cell surface trafficking of annexin A2 independent of clathrin [72]. Phosphorylation of annexin A2 at Tyr-23 was shown to be essential for stabilization of lipid raft complexes and its subsequent association with the endosomal system.

Exosomes are small membrane (30–100 nm) vesicles secreted by cells of hematopoietic origin and epithelial cells by fusion of the late Multivesicular Endosomes (MVE) with the cell membrane [127,128]. Very early studies using mass spectrometry had identified annexin A2 as an important component of exosomes in dendritic cells [128], but precise mechanism of secretion of annexin A2 was not established. Valapala and Viswanatha demonstrated that annexin A2 was secreted in exosomes in a raft associated manner [72]. They showed that in the ionophore stimulated NIH3T3 cells, annexin A2 is secreted in the exosomes through MVEs and these can then be transferred from one cell to another. Although this study did not highlight the importance of S100A10 in exosomal secretion of annexin A2, recent studies by Fang *et al.*[129] suggest that annexin A2 is associated to the exosomal secretion pathway in a S100A10 dependent manner in lung epithelial cells. In this investigation, stimulation of lung epithelial cells by IFN-γ resulted in increased surface translocation of annexin A2 and S100A10 primarily by enhanced expression of S100A10 via the JAK2/STAT1 pathway. They observed increased secretion of annexin A2 in the exosomes in IFN-γ treated cells, which was markedly reduced in cells where S100A10 expression was silenced. Together these studies delineate mechanism of annexin A2 secretion into exosomes. Exosomes function in the immune system and also promote intercellular signaling [127]. Tumor-derived exosomes have also been identified and are thought to promote tumor cell growth and metastasis [130]. Interestingly annexin A2 has been identified in melanoma exosomes using proteomics [131,132]. It was suggested that exosomal annexin A2 could serve as a diagnostic tool and as a biomarker in cancer progression and prognosis.

Although S100A10 may not be required for annexin A2-mediated endosomal association, S100A10 plays an important role in mobilizing annexin A2 for various functions in membrane dynamics. It mediates the translocation of annexin A2 to the cell surface in some cell types [71]. It also targets annexin A2 to the cortical cytoskeleton and enhances annexin A2 dependent F-actin bundling [133–135]. In a more recent investigation, Sylvette Chasserol-Golaz’s group has shown that S100A10 physically interacts with the VAMP2 protein *in vitro* and at the plasma membrane in adrenergic chromaffin cells. S100A10 also forms clusters along with VAMP2 and syntaxin to which annexin A2 translocates [136]. Annexin A2 translocates to the membrane and forms a tetrameric complex with S100A10 in secretagogue-stimulated cells. It is suggested that AIIt plays an important role in exocytosis as a potential binding partner for SNARE proteins and also by targeting these proteins to membranes.

### 8.3. Epithelial and Endothelial Cell Polarity

In response to external stimuli, annexin A2 is capable of promoting epithelial cell polarity by orchestrating two processes, namely cell-cell adhesion and formation of adherens junctions. Since annexin A2 regulates actin dynamics, and weakly interacts with PtdIns(3,4,5)P3, it is plausible to consider annexin A2 as an important modulator of cell-cell adhesion. In fact, annexin A2 recruits and regulates the activation of Rho and Rac1 GTPases [137,138] both of which are essential for initiating actin cytoskeleton reorganization during cell-cell adhesion [139,140]. Additionally, it interacts with AHNAK and recruits it to cholesterol-rich microdomains which are also essential for regulation of actin cytoskeleton [45].

AIIt complex also functions in adherens junction formation in epithelial [141] and endothelial cells [142] by its association with epithelial E-cadherin and endothelial VE-cadherin. Annexin A2 depletion ablates E-cadherin recruitment to the adherens junction without affecting the formation of tight junctions. Loss annexin A2 expression in endothelial cells results in instability of adherens junctions and absence of VE-cadherin at these junctions. Extracellular annexin A2-S100A10 complexes are also shown to be involved in cell membrane bridging and tight junction formation [143].

### 8.4. mRNA Binding

Annexin A2 is one of several proteins found in mRNP (ribonucleoprotein) complexes and may function as a nuclear scaffold protein for recruitment of other proteins. The function of annexin A2 in binding mRNA was first demonstrated in rous sarcoma virus transformed and normal cells, where 10%–15% of annexin A2 was shown to associate with ribonucleoparticles (RNPs) [101]. Immunoprecipitation of annexin A2 from UV-irradiated cultured cells revealed an association of annexin A2 with RNA as a part of RNP complex. Subsequently studies have shown that annexin A2 is found in the nucleus in association with Z-DNA [144], and as a part of primer recognition complex which stimulates DNA polymerase α activity [102,104]. Moreover the cytoskeleton-associated annexin A2 was shown to localize with mRNA complexes in the cytoskeleton [145]. The presence of NES in annexin A2 [33] further suggests that it could play an important role in RNA binding, transport and translation. Comprehensive studies in our laboratory further established that annexin A2 binding to mRNA is a calcium-dependent event. In fact, annexin A2 specifically binds to oncogenic c-myc mRNA and regulates its translation, as expression of annexin A2 showed enhanced levels of c-myc protein [103]. Interestingly, this mRNA binding function was observed only for monomeric annexin A2. By mapping the mRNA binding site of annexin A2 to the helices C-D in its domain IV, Vedeler’s group recognized that this motif is unique among the previously identified RNA binding motifs (such as the RNP motif, the arginine-rich motif, the RGG box, the KH motif, the double stranded RNA-binding motif, and the zinc finger-knuckle motif) [146]. Specifically, the positively charged polar residues in this domain that are easily accessible and solvent exposed are involved in RNA binding. The initial interactions with RNA are suggested to be non-specific electrostatic interactions between lysine residues in annexin A2 and the negatively charged phosphate residues in the mRNA backbone. Further stabilization of the interaction is promoted by the conformation change in annexin A2 upon to calcium-binding [103,146]. The annexin A2 binding site on the mRNA of c-myc and annexin A2 involves an 80–100 nucleotide sequence in the 3′-UTR region of the mRNA. The consensus sequence for mRNA binding is 5′-AA(C/G)(A/U)G, which is repeated three times in annexin A2 mRNA and only once in c-myc mRNA [64,147]. Moreover the binding not only depends on the consensus sequence but also on the presence of higher order secondary structure of the mRNA such as a pseudoknot structure preceding the consensus sequence. Post-translational modification of annexin A2 affects its RNA binding. Annexin A2 heterotetramer failed to bind to RNA when it was bound to phospholipids, whereas F-actin binding did not interfere with mRNA binding. F-actin-mRNA interaction is well described functioning to anchor the mRNA in the cytoskeleton during the final stages of mRNA transport [148]. Thus, annexin A2 can act as a scaffolding protein for F-actin mediated stabilization of mRNA transcripts in the cytoskeleton hence playing a role in post-transcriptional regulation of gene expression.

### 8.5. Plasminogen Receptor—S100A10, and Not Annexin A2, Binds Plasminogen and tPA and Regulates Plasmin Generation

Plasminogen receptors play a crucial role in regulating plasmin activation on the cell surface. The inactive zymogen plasminogen is converted to the active serine protease plasmin by the plasminogen activators, tissue plasminogen activator (tPA) and urokinase plasminogen activators (uPA). The presence of plasminogen activator inhibitors (PAI-1 and PAI-2) and plasmin inhibitor α1-antiplasmin regulates plasmin activity to prevent inappropriate proteolysis on the cell surface. Plasmin activation is involved in many physiological and pathological processes including fibrinolysis and tumor cell invasion. This is mediated at the surface of the endothelial cells and tumor cells which involves the dissociation of extracellular matrix and basement membrane The rate of plasmin production is significantly accelerated by the presence of plasminogen receptors on the cell surface, primarily by binding plasminogen and also by co-localizing the plasminogen activators tPA and uPA [149–151].

One key feature of majority of plasminogen receptors is the presence of a carboxyl-terminal lysine residue. This lysine residue interacts with the lysine-binding (kringle) domains of tPA and plasminogen and thereby participates in plasminogen binding and activation at the cell surface [152]. Annexin A2 was originally identified as a plasminogen receptor whose binding to plasminogen was blocked by pre-treatment with carboxypeptidase B [153]. Since carboxypeptidase B removes carboxyl-terminal lysines and annexin A2 does not possess a carboxyl-terminal lysine, it was speculated that a new carboxyl-terminal lysine was generated by a post-translational event, namely, through cleavage at Lys-307-Arg-308 by an unidentified protease at the cell surface [153]. Nearly twenty years later, the presence of a proteolytically processed annexin A2 has not been demonstrated at the cell surface and rigorous studies, while successful at identifying full-length annexin A2 at the cell surface, have been unsuccessful in the identification of the proteolyzed annexin A2 [18,19,21]. For example, we have demonstrated that only the full length annexin A2 is present on the surface of thioglycollate-elicited macrophages. This experiment involved injecting the peritoneal cavity of mice with thioglycollate, an inflammatory stimulus. This was followed by collecting the macrophages that migrated from the blood to the peritoneal cavity in response to the inflammatory stimulus. Since in order for macrophages to transverse tissue barriers they must generate plasmin, the thioglycollate model system is considered the gold standard for examining the role of proteases in cell migration *in vivo*. Examination of the cell surface annexin A2 of the thioglycollate elicited macrophages revealed that annexin A2 was not proteolysed. This established that the proposed annexin A2 post-translational proteolytic cleavage event does not occur *in vivo*[154]. The annexin A2 protein used in previous experiments was eluted from polyacrylamide gels as a 40 kDa band and the possibility that this annexin A2 was denatured and subsequent results artifactual, must be now considered. Consequently, it has been clearly established that intact annexin A2 does not bind plasminogen [155,156], and annexin A2 is not cleaved *in vivo*. It is now accepted that annexin A2 is not a plasminogen receptor.

The role of annexin A2 as a tPA-binding protein is more perplexing as it was originally demonstrated that the Cys-8 residue of annexin A2 formed the binding site for tPA. However, subsequent studies that utilized mutagenesis of the amino acid residues in the vicinity of Cys-8 demonstrated that the residues that flanked Cys-8 had no influence on the binding of tPA to annexin A2. Simply interpreted, these experiments establish that tPA forms a covalent disulfide bond with annexin A2 *in vitro*[157]. However, the binding of tPA to cells is known to be reversible and dependent on a carboxyl-terminal lysine on the tPA receptor [158,159], which contrasts with the formation of an irreversible covalent disulfide bond between tPA and annexin A2. Furthermore, the interaction between tPA and its binding sites on the cell surface are known to involve the finger and kringle-2 domains [160–162]. In fact, the kringle-2 domain of tPA binds to lysine Sepharose and to plasminogen activation cofactors (e.g., fibrin, denatured albumin) [163,164]. This interaction can blocked by the carboxyl-terminal lysine mimetic, ɛ-aminocaproic acid [163] indicating that this domain is key in the interaction of tPA with its cellular receptors. In addition, tPA only contains one free thiol which is present in the EGF domain which is capable of forming a disulfide bond with annexin A2 [165]. However, the EGF domain of tPA does not participate in binding to the cell surface and it is therefore most unlikely that annexin A2 forms a disulphide linkage with this residue *in vivo*. Lastly, a recent study has shown that tPA binding to the cell surface of endothelial cells does not co-localize with annexin A2, suggesting that annexin A2 is not a tPA receptor on these cells [162].

As discussed previously, annexin A2 can exist in the cells as a monomer, or a heterotetramer in complex with S100A10. Interestingly in cell types such as endothelial, epithelial, and MDCK cells most of the annexin A2 is present on the cell surface as the heterotetramer form AIIt [107,166,167]. Moreover when annexin A2 was specifically down-regulated in MDCK cells by siRNA, both annexin A2 and S100A10 protein levels were significantly reduced in these cells [45]. This is consistent with the fact that annexin A2 protein has been shown to regulate the expression of S100A10 by post-translational modification, primarily by stabilizing S100A10 on the cell surface [13–15,168,169]. Interestingly, down-regulation of S100A10 by siRNA did not result in decrease in annexin A2 protein expression [155,170]. More recently our laboratory has used human microvascular endothelial cell line (TIME – telomerase immortalized microvascular endothelial cell), to deplete protein levels of both annexin A2 and S100A10 by shRNA. S100A10-depleted cells showed a significant decrease in plasminogen binding and plasmin generation, in the presence of identical cell surface level of annexin A2. Depletion of annexin A2 in TIME cells resulted in similar losses in plasminogen binding and plasmin generation as in S100A10-depleted cells [171]. This is likely due to the loss of S100A10 in cells depleted of annexin A2. Homozygous annexin A2-null mice show increased deposition of fibrin in the blood vessels and incomplete clearance of injury induced arterial thrombi [172]. These authors suggested that annexin A2 played an important role in fibrinolysis and plasmin generation. However, as discussed these studies did not take into account the critical fact that annexin A2 knock-out also results in concomitant loss of S100A10 in these mice. In contrast, our laboratory observed enhanced accumulation of fibrin in the tissues of the S100A10-null mice. These S100A10-null mice had significantly lower rates of fibrinolysis suggesting a decreased level of plasmin generation by the microvasculature that was devoid of S100A10 but that contained wild type levels of annexin A2 [171].

Several investigators have previously reported that annexin A2 is required for plasmin generation and matrix invasion by macrophages [173,174]. However, we have recently established that S100A10, and not annexin A2, is responsible for not only promoting the reported macrophage plasminogen-mediated matrix invasion and degradation but also the infiltration of tumor-promoting macrophages into tumor sites [20,154]. Moreover, we have shown that enhanced generation of plasmin exhibited by promyelocytic leukemic cells (NB4), which was originally ascribed to annexin A2 [173], was in fact misinterpreted and can now be attributed to S100A10.

**Figure 3:**
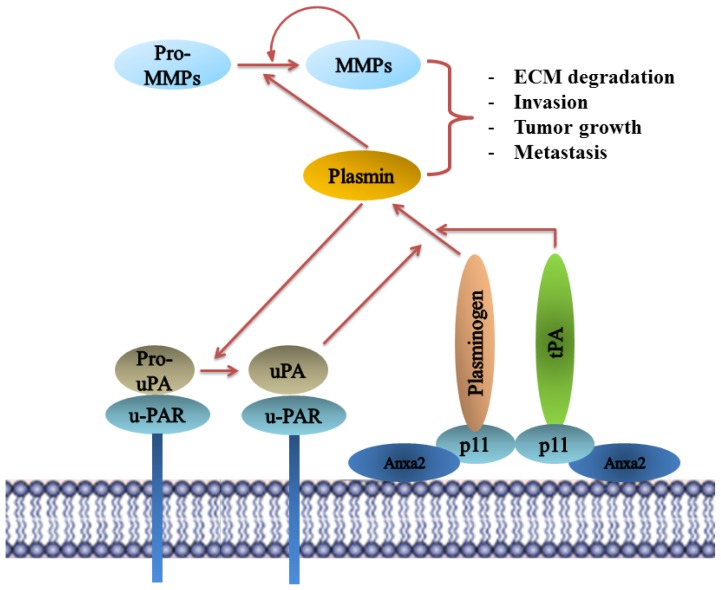
Experimental model of plasmin regulation by cell surface annexin A2 and S100A10. The heterotetrameric complex consists of two copies of annexin A2 and one copy of the S100A10 dimer. AIIt binds the tissue-plasminogen activator tPA and plasminogen at the carboxyl-terminal lysine residue of the S100A10 subunit. The annexin A2 subunit does not bind tPA or plasminogen but serves as cell surface receptor for S100A10. The urokinase-plasminogen activator is bound to its receptor (uPAR) and forms the uPA/uPAR complex that colocalizes with AIIt. The co-localization of the plasminogen activators and plasminogen by AIIt results in accelerated cleavage of plasminogen into plasmin. Plasmin activates pro-MMPs (matrix metallo-proteases) into active MMPs and further activates pro-uPA into active uPA.

Thus rigorous scientific analysis suggests that annexin A2 plays an important role in regulating plasmin activity not by acting as a direct plasminogen receptor but by stabilizing and transporting S100A10 to the cell surface where it serves as a cell surface receptor for S100A10 (Figure 3).

## 9. Role of Annexin A2 in Diseases

As described, annexin A2 is a multifunctional protein involved in gene regulation, cellular transformation, regulating membrane dynamics, cytoskeletal re-arrangement, and fibrinolysis. Thus, it is not surprising that it has been implicated in a number of diseases. Here, we will describe in detail the role of annexin A2 in cancer progression, inflammation and anti-phospholipid syndrome (APS).

### 9.1. Annexin A2 in Cancer Progression

Accumulating evidence delineates a correlation between the deregulation of annexin A2 expression and tumorigenesis in many cancers but disregard the possible role of S100A10. Nevertheless, annexin A2 has been suggested to be capable of modulating key events in tumor progression mainly those involving invasion, metastasis, and drug resistance (Table 1). In fact, annexin A2 is proposed as a potential diagnostic/prognostic marker for prediction of tumor malignancy, metastatic recurrence and patient survival. Annexin A2 expression in cancer is relatively paradoxical as it may act as a tumor suppressor or as an oncogene depending on the type of cancer. This not only reflects the lack of complete understanding of annexin A2 regulation in tumors but also the fact that annexin A2 deregulation is probably affecting gene expression or is simply a bystander effect as a consequence of change in expression of major cancer-regulating genes. Gou *et al.*[175] reported that annexin A2 depletion by shRNA results in the deregulation of expression of 61 genes in type-II alveolar cells. In contrast, microarray analysis by our laboratory showed that S100A10 depletion only affected S100A10 mRNA levels and has no effect on gene expression (unpublished data). Since annexin A2 is a redox protein it is possible that oncogenic changes in annexin A2 may be related to changes in cellular redox status, particularly since oncogenes are known to increase intracellular levels of H_2_O_2_, which in turn is known to increase annexin A2 levels [99]. On the other hand, S100A10 acts as the effector molecule whose activity can be determined by the levels of expression of annexin A2. Since S100A10 is a well-documented plasminogen receptor it is likely that oncogenic stimulation of annexin A2 levels also serve to increase S100A10 levels. This then has a direct impact on cell surface plasmin generation and proteolytic activity of cancer cells

Many studies have attributed the role of plasmin generation and cancer cell invasion to annexin A2 (discussed next). These studies must be re-examined in light of the recent findings about the relationship between annexin A2 and its binding partner S100A10. Nonetheless, annexin A2 can be an indirect contributor to plasmin generation as it stabilizes S100A10 which in turn directly participates in binding to tPA and plasminogen and enhancing plasminogen activation. Once cleaved, plasminogen is converted into its active proteolytic form plasmin. Plasmin not only mediates the hydrolysis and remodeling of the extracellular matrix (ECM) but also activates other key matrix metallo-proteases (MMPs) such as MMP-9 and cathepsin B [176,177] (Figure 3). Importantly, matrix remodeling is an essential step in tumor cell mobilization and often exploited by tumors to promote invasion. Plasmin-mediated ECM proteolysis allows tumor cell invasion into surrounding tissues, basement membrane degradation, and transmigration into circulation leading to distant-site metastasis. MMP-9 activation also contributes to tumor angiogenesis by releasing sequestered VEGF from the ECM [178].

The role of annexin A2 in cellular transformation was first identified by Erikson and Erikson using fibroblasts which were transformed by the avian sarcoma virus-transforming gene product (pp60src). The transformation was mediated by pp60src-specific phosphorylation of annexin A2 [10]. In 1993, Chaing *et al.*[179] further elucidated that annexin A2 expression is regulated by the cell cycle suggesting a potential role of annexin A2 in cell proliferation. Both annexin A2 mRNA and protein levels increased in G1 reaching a maximum during early S phase.

### 9.2. Annexin A2-S100A10 Complex and Not Monomeric Annexin A2 Mediates Invasion, Metastasis and Drug Resistance in Breast Cancer and May Act as Pathological Predictor

Annexin A2 is consistently over-expressed in patients with both invasive ductal mammary carcinoma and ductal carcinoma in situ (DCIS). In contrast, it is undetectable in normal and hyperplastic breast ductal epithelium [180] suggesting a role of annexin A2 in malignancy and tumor invasiveness.

Accumulating evidence suggest that invasiveness of breast cancer is maintained through enhanced plasmin generation. Treatment with angiostatin inhibits breast cancer growth and lung metastasis formation via binding to annexin A2 and inhibiting annexin A2-mediated plasmin generation [181]. In fact, annexin A2 mediates tPA-dependent plasmin generation (probably through S100A10) and promotes the *in vitro* migration of MDA-MB-231 breast cancer cells [182]. The AIIt tetramer is over-expressed on the surface of these cells which may justify their highly invasive nature. Annexin A2 expression also correlated with increased recruitment of inflammatory cells into tumor sites and with elevated neoangiogenic activity in breast cancer patients [182]. Interestingly, S100A10 expression on tumor-associated macrophages (TAMs) is required for their recruitment to primary tumor sites in a lewis lung carcinoma (LLC) mouse model. In fact, S100A10-null mice develop smaller and less angiogenic tumors due to failure of recruitment of these macrophages [20]. This suggests that the reported role of annexin A2 in the early activation of the “angiogenic switch” is probably mediated through S100A10 and not annexin A2. In addition to its interaction with S100A10, annexin A2 also directly interacts with the glycoprotein tenascin C (TNC). TNC supports the establishment of the pre-metastatic niches and promotes breast cancer metastasis into lungs. This direct annexin A2-TNC interaction at metastatic sites might also drive metastatic cell proliferation through annexin A2 signaling [183]. The pathway of annexin A2-mediated cell proliferation remains largely unclear.

Annexin A2 is also potentially involved in promoting breast cancer resistance to chemotherapeutic anthracyclines and taxanes. Quantitative proteomic analysis revealed 5.4-fold and 1.8-fold up-regulation of annexin A2 in doxorubicin- and paclitaxel- resistant MCF-7 cells [184]. Reduction in annexin A2 expression in doxorubicin-resistant MCF-7 cells led to slower proliferation and significantly decreases their invasive capacity [184]. Notably, annexin A2 reduction failed to restore doxorubicin sensitivity suggesting that annexin A2 is not the sole contributor to resistance in this cell line.

Clinically, annexin A2 may act as a predictor of pathological response to neoadjuvant chemotherapy in advanced breast cancer patients. Pre-treatment annexin A2-positive patients are predicted to have poor pathological response upon chemotherapy [100]

### 9.3. Annexin A2 Promotes Invasion and Metastasis in Pancreatic Ductal Adenocarcinoma (PDAC) and Is Predictive of Post-Operative Recurrence and Patient Survival

Pancreatic ductal adenocarcinoma (PDAC) is responsible for over 90% of pancreatic cancers with a 5-year survival rate of 5%. The high mortality rate of PDAC is mainly caused by the enhanced metastatic capacity of the disease combined with lack of early diagnosis. Proteomic analysis of PDAC patient samples revealed that annexin A2 is overexpressed in 88.6% of tumor tissues and only 34.2% of non-tumorous pancreatic tissues. Immunohistochemical (IHC) analysis of early pancreatic lesions showed that annexin A2 is only found in 36% of PanIN-1A (Pancreatic intraepithelial neoplasia) and 19% of PanIN-1B non-invasive lesions. In contrast, annexin A2 was expressed on more than 80% of the more invasive lesions (PanIN-2 and PanIN-3) suggesting that annexin A2 might be a contributor to the progressive invasiveness of PDAC [185]. However, this increased invasiveness is most likely to be mediated by S100A10, and not annexin A2, since S100A10 expression is also highly up-regulated in PanIN lesions [186]. Furthermore, in the invasive lesions, annexin A2 is primarily localized to the cell surface with a weak cytoplasmic signal predicting the involvement of surface plasmin-mediated invasion of PDAC [187,188]. Annexin A2 blockade by shRNA or anti-annexin A2 antibodies inhibited invasion of pancreatic cell lines (human Panc 10.05 and mouse Panc02) and reduced liver metastasis in a PDAC mouse model [83]. The effect of annexin A2 depletion on S100A10 expression is not reported in this paper and is probably responsible for the loss of invasiveness in these cell lines. The cell surface localization of annexin A2 and its invasion-promoting function require the phosphorylation of the Tyr-23 residue [83]. This phosphorylation event is likely to be critical for S100A10 stabilization and expression on the cell surface.

Epithelial mesenchymal transition (EMT) is a process through which epithelial cells acquire a motile phenotype which is essential for embryonic development and tissue remodeling. Tumors exploit the plasticity of epithelial cells to undergo EMT in order to promote invasion and metastasis. Rescher *et al.*[87] identified that Tyr-23 phosphorylation of annexin A2 is critical for the active remodeling of membrane-associated actin cytoskeleton through Rho/ROCK signaling pathways [87]. This remodeling allows tumor cell motility and adhesion both of which are characteristic of EMT. Expectedly, shRNA blockade of annexin A2 results in inhibition of TGFβ-induced Rho-mediated EMT of PDAC cells [83].

In addition to its binding partner S100A10, annexin A2 interacts with multiple splice variants of TNC. The latter is a hexameric anti-adhesive matrix glycoprotein that interacts with other ECM proteins (fibronectin), surface receptors (annexin A2, cell adhesion integrin) and syndecan membrane proteoglycans all of which are reported to be overexpressed in pancreatic cancer [183,185]. Annexin A2-TNC interaction promotes mitogenesis, migration, and loss of focal adhesion in endothelial cells that can be blocked by anti-annexin A2 antibodies [189]. Both annexin A2 and TNC were found to be over-expressed and co-localized in high-grade PanIN lesions and PDAC [185] suggesting a potential role of their interaction in advanced stages of pancreatic cancer.

Annexin A2 has been clinically implicated with recurrent disease in patients with advanced PDAC. In fact, high levels of annexin A2 correlate with pancreatic cancer recurrence in post-operative patients who were previously treated with gemcitabine adjuvant therapy [190]. Patients with high levels of annexin A2 expression also have a worse disease-free survival than those with low annexin A2 levels [83]. These studies support annexin A2 as a prognostic indicator of pancreatic cancer recurrence and patient survival.

### 9.4. Annexin A2 Is a Metastatic Marker in Renal Cell Carcinoma (RCC)

Renal cell carcinoma (RCC) is the most common kidney cancer and accounts for 3% of all adult cancers. It represents a group of heterogeneous tumors with distinct biological characteristics of which clear-cell renal carcinoma (ccRCC) is the most common (80% of all RCCs). RT-qPCR and immunohistochemistry analysis showed that annexin A2 is up-regulated in RCC tissues at both the mRNA and protein levels compared to normal renal tubule tissues [191]. In fact, both members of the AIIt tetramer (annexin A2 and S100A10) were up-regulated in RCC patient tissues suggesting that AIIt expression, and not only annexin A2, can be a potential diagnostic marker in these patients. At this point, the role of S100A10 in RCC is yet to be investigated.

Interestingly, the up-regulation of annexin A2 in RCC is not limited to primary tumor sites. Its expression is extended and amplified in metastatic sites of ccRCC patients. Immunohistochemical analysis of patient ccRCC tissues revealed that annexin A2 was over-expressed in 47.4% of primary tumors and 87.5% of metastatic tumors. The 5-year metastasis-free survival rate of patients with high annexin A2 expression was significantly lower than those with no annexin A2 expression [192]. These results suggest that annexin A2 is a novel predictor of the metastatic potential of ccRCC.

### 9.5. Annexin A2 Expression Correlates with TNC Expression and Is a Potential Prognostic Marker of Advanced Colorectal Carcinoma (CRC)

Colorectal cancer (CRC) is the third most common cancer in men and women. Annexin A2 is up-regulated in 29.5% of colorectal cancer patient samples and this up-regulation correlated with tumor size, advanced histology, and depth of invasion and pTNM stage (pathological tumor-node-metastasis). Annexin A2 over-expression also correlated with TNC expression in colorectal tissues [193]. Tissue microarrays and proteome analysis showed that up-regulated annexin A2 expression also predicted higher lymph node metastasis (LNM) in colorectal cancer patients [194]. Interestingly, Zhang *et al.*[195] identified S100A10 as the major contributor to plasminogen binding, plasmin generation and subsequent invasiveness of Colo 222 colorectal cancer cells, cells which do not express surface annexin A2. Moreover, S100A10 expression correlated with tumor recurrence in stage II and III colon cancer patients treated with 5-fluoruracil [196]. Importantly, it therefore appears that the invasiveness of cancer cells observed in colorectal cancer is potentially maintained through the increased stabilization of S100A10 (and not annexin A2) as invasion can also be mediated through an annexin A2-independent manner as observed in the Colo 222 cells.

### 9.6. Annexin A2 Is a Differential Diagnostic Tissue and Serum Marker in Hepatocellular Carcinoma (HCC)

The up-regulation of annexin A2 in HCC was first described by Frohlich *et al.*[197] and was associated with malignant transformation of hepatocytes and not with liver tissue regeneration. The expression of annexin A2 in hepatocellular carcinoma is up-regulated at the transcriptional and translational level [197]. Interestingly, the non-tumorous cirrhotic tissues expressed relatively high levels compared to the adjacent tumorous tissues suggesting that annexin A2 up-regulation is potentially a by-product of inflammation rather than oncogenic transformation per se. This is further supported by the report that plasmin-mediated cleavage of annexin A2 activates intracellular pathways which result in the activation of MAP kinases and NF-κB nuclear translocation. NF-κB activates the expression of pro-inflammatory molecules such as IL-1, IL-6 and TNFα [198].

Annexin A2 staining was intense in moderately and poorly differentiated tumors and not in well-differentiated tumors [199]. This is consistent with the function of annexin A2 in cellular differentiation and suggests annexin A2 as a possible determinant of histological grade in HCC patients. Using annexin A2 expression as a biomarker can improve the sensitivity and specificity of the de-facto 3-marker IHC panel of HSP7 (heat shock protein 70), GS (glutamine synthase), GPC-3 (glypican-3) used to differentiate early stage well differentiated HCC from normal liver mitogenesis. In fact, combinations of annexin A2-GPC3 and annexin A2-GS performed better than the 3-marker combination [200] suggesting that adding annexin A2 as diagnostic marker increases the reliability of HCC diagnosis in liver biopsies. Recent microarray analysis of HCC patient samples revealed multiple hotspots that were linked to the Akt/NF-κB signaling pathways. These gene expression hotspots included annexin A2, S100A10 and the diagnostic HCC marker GPC-3 [201] indicating that annexin A2 and S100A10 expression at the protein levels may promote hepatocarcinogenesis through the activated Akt pathway. Serum levels of annexin A2 are also elevated in early stage HCC patients which are AFP-negative [202]. Alpha-fetoprotein (AFP) is a widely used marker for surveillance and early detection of hepatic cancers. The results indicate that annexin A2 serum level is a better diagnostic marker than the traditional AFP screening, although combining both markers yielded the most accurate diagnosis with 87.4% accuracy [202].

### 9.7. Annexin A2 May Promote Hyperfibrinolysis and Acute Bleeding in Acute Promyelocytic Leukemia (APL)

Bleeding in acute promyelocytic leukemia (APL) is caused by intravascular coagulation and hyperfibrinolysis. The latter is mediated through enhanced plasmin generation which results in the breakdown of fibrin. APL patients express higher levels of annexin A2 than other leukemic patients. PML-RARα-positive APL cells have enhanced cell surface tPA-dependent plasmin generation which can be inhibited by anti-annexin A2 antibodies [203]. Moreover, annexin A2 depletion in NB4 cells decreased plasmin generation by 60% while its expression in HL-60 cells (APL cell line with low endogenous annexin A2) resulted in a 5-fold increase in plasmin generation [204]. However, we have recently demonstrated that induced expression of PML-RARα increases the expression of cell surface S100A10 which caused a dramatic increase in fibrinolytic activity of these NB4 cells [205]. This clearly indicates that S100A10, and not annexin A2, is the actual regulator of plasmin-mediated fibrinolysis in these cells and that PML-RARα-mediated S100A10 up-regulation is likely to be responsible for the bleeding symptoms in APL patients.

The arsenic trioxide (ATO) or all-trans retinoic acid (ATRA) treatments of APL patients are currently used to induce terminal differentiation of leukemic promyelocytes which potentially leads to complete remission [204,206]. Both treatments induce rapid amelioration of APL-associated bleeding through the down-regulation of annexin A2 [207,208] and more importantly S100A10 [205]. This down-regulation reduces plasmin generation and aids in resolution of coagulopathy. Most studies have reported that annexin A2 expression is elevated in many cancers and can directly correlate with clinical outcome. However, annexin A2 down-regulation have been reported in some cancers (prostate cancer and head and neck cancers), yet some of these results remain conflicting mainly due to difference in methodologies.

### 9.8. Annexin A2 Expression Presents Conflicting Results in Prostate Cancer

Annexin A2 mRNA expression is often lost in prostate cancer specimens when compared to normal prostate tissues. In fact, immunohistochemistry of prostate cancer patient samples showed a loss in expression of annexin A2 and its binding partner S100A10 in all patients [168,209]. Both proteins are also lost in 65% of prostate intraepithelial neoplasia (a prostate cancer precursor) [168]. Microarray analysis revealed that annexin A2 (along with annexin A1, A4, A7 and A11) was significantly down-regulated in metastatic androgen-independent prostate cancer patients when compared to hormone-naive prostate cancers and non-cancerous prostate tissues [210]. This suggests that the loss of annexin A2 is potentially required for the acquisition of androgen refraction which ultimately leads to the development of metastatic prostate cancer. Liu *et al.*[84] demonstrated that annexin A2 is abundantly expressed in five strains of normal human prostate (NHP 1-5) epithelial cells while reduced/lost in seven prostate cancer cell lines (PPC-1, MDA PCa 2b, LNCaP, C4-2, C5, PC3 and DU-145). Expectedly, retroviral re-expression of annexin A2 in prostate cancer cells (DU-145 and LNCaP) with low/no endogenous annexin A2 significantly inhibited migration of both cell lines [84]. This loss in migration capabilities is consistent with the established role of annexin A2 in cellular motility [109]. It also is suggested that reduction in annexin A2 expression is maintained epigenetically through DNA hypermethylation since treating LNCaP cells with DNA methylation inhibitors (e.g., 5-aza-deoxycytidine) restored expression of annexin A2 [84].

The aforementioned studies reported that the reduction of annexin A2 expression correlated with high grade metastatic prostate cancers. However, Banerjee *et al.*[211] observed contradicting results when comparing annexin A2 expression in prostate carcinoma patients between USA and India. Interestingly, the results revealed that 25% of high grade prostate cancer US patients show intense focal membrane staining of annexin A2 which was not found in Indian patients with similar grades. In addition, the study observed that the DU-145 prostate cancer cell line expressed annexin A2 while LNCaP did which contradicts the Liu *et al.* observations [84]. More recently, a retrospective study of patient samples with non-metastatic prostate cancer revealed that all annexin A2-positive tumors (*n* = 40) relapsed compared to only half in annexin A2-negative tumors. Furthermore, shRNA knockdown of annexin A2 in DU-145 cells inhibited their growth in culture without affecting their invasiveness [212]. In addition, annexin A2 expressed on osteoblasts facilitates the homing and migration of annexin A2 receptor bearing prostate tumors cells contributing to bone metastasis. Annexin A2 can also facilitate the growth of prostate cancer cells *in vitro* through activation of the MAPK pathway [213]. Unlike Liu *et al*. and Chetcuti *et al.*, these studies delineate a positive correlation between annexin A2 and prostate cancer malignancy and further contribute to the uncertainty of annexin A2 role in prostate cancer.

### 9.9. Annexin A2 Expression Is Prognostic of Histological Grade and Metastasis in Most Head and Neck Cancers

The down-regulation of annexin A2 has been implicated in head and neck cancers primarily in esophageal squamous cell carcinoma (ESCC), nasopharyngeal carcinoma (NPC), and head and neck squamous cell carcinoma (HNSCC). RT-PCR and immunohistochemical analysis of ESCC patient samples obtained from an area with high incidence of esophageal cancer (Henan province, China) showed significant decrease in annexin A2 expression in these patients compared to normal esophageal epithelium. Annexin A2 also negatively correlated with the differentiation status of ESCC tumors with less differentiated malignant tumors having the lowest annexin A2 levels [214] suggesting a role of annexin A2 in ESCC malignant progression.

Proteomic analysis of nasopharyngeal carcinoma (NPC) revealed a consistent reduction in annexin A2 expression in all NPC patients and cell lines when compared with normal nasopharyngeal epithelium. Annexin A2 down-regulation in these patients is prognostic and correlated with lymph node metastasis [215]. Using immunochemistry and RT-PCR, Pena-Alonso *et al.*[216] reported that annexin A2 is down-regulated in head and neck squamous cell carcinoma (HNSCC) compared to normal basal epithelial cells and this reduction correlated with less differentiated tumors and nodal metastasis. Despite these results, Wu *et al.*[217] identified an up-regulation of annexin A2 in HNSCC cells which were derived from metastatic tumors and not in cells derived from primary tumors (using mass spectrometry and 2D-gel electrophoresis). This contradicts with the previous studies by Qi *et al.*[214] which support annexin A2 down-regulation in primary tumors. The difference in annexin A2 expression between the two groups is thought to be due technical differences as mass spectrometry is highly sensitive and may identify annexin A2 protein modifications that cannot be detected using immunohistochemistry or RT-PCR. Whether annexin A2 is up- or down-regulated, the mechanism behind its transcriptional and translational regulation is yet to be fully established.

### 9.10. Annexin A2 Is a Promising Therapeutic Target in Cancer

Targeting annexin A2 with antibodies has been routinely used by researchers to reduce tumor progression and metastasis. In a study by Zheng *et al.*[83], anti-annexin A2 antibodies reduced metastasis and prolonged the survival of a PDAC mouse model. Anti-annexin A2 antibodies or sera from PDA patients post vaccination also inhibited invasion of PDAC cells *in vitro*[83]. Anti-annexin A2 antibodies have been also effective to inhibit tumor growth in a mouse model of LLC and reduce plasmin generation *in vitro*[180].

In addition to specific antibodies, other molecules have been proposed for direct targeting of annexin A2. The anti-angiogenic molecule angiostatin inhibits annexin A2 by binding to its lysine domain [181] and reduces plasmin generation in LLC cells [180]. Synthetic inhibitors have been also developed to target annexin A2. TM601 is a synthetic inhibitor derived from chlorotoxin and acts as an anti-angiogenic molecule through direct binding to annexin A2. TM601 also inhibits tPA-mediated plasmin generation and *in vitro* migration of pancreatic (Panc-1), glioma (U87-MG, U373-MG), and lung carcinoma (A549) cells [218]. PLGA (poly lactide-co-glycolide) nanoparticles loaded with annexin A2 shRNA vector can mediate long-term silencing of annexin A2 and are able to reduce tumor growth in xenograft models of prostate cancer [219]. These studies reveal that annexin A2-specific targeting has promising therapeutic benefits in animal models but is yet to be elucidated in human trials.

### 9.11. Role of the Extracellular Annexin A2-S100A10 Complex in Inflammation

**Figure 4:**
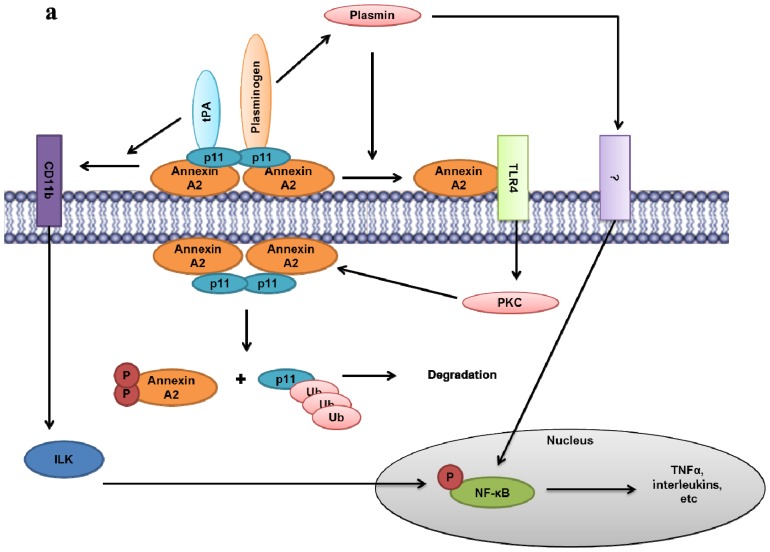

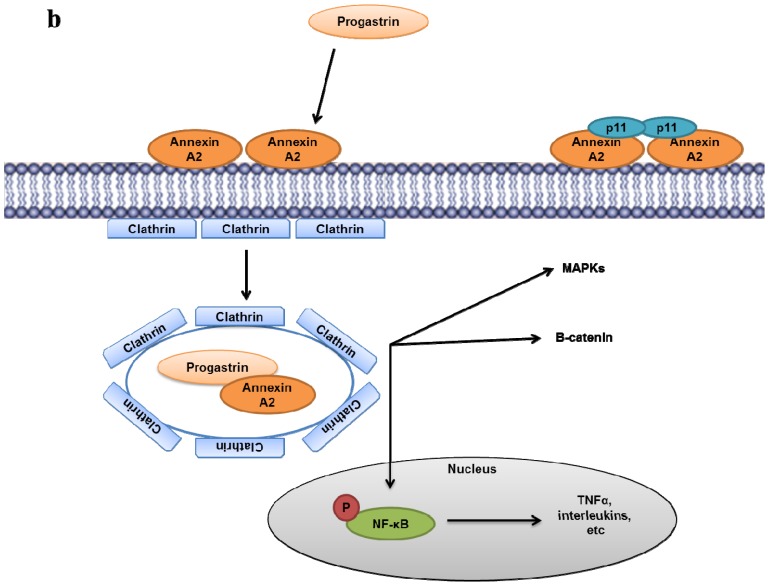
Experimental model of inflammatory pathway regulation by the annexin A2-S100A10 heterotetramer. (**a**) Plasmin generated by the annexin A2-S100A10 heterotetramer (AIIt) induces the co-localization of membrane-bound annexin A2 and Toll-Like receptor 4 (TLR4), which causes PKC-mediated phosphorylation of cytoplasmic annexin A2 at serines 11 and 25. The phosphorylation of annexin A2 disassembles AIIt allowing the ubiquitation and degradation of S100A10. In addition, tissue-plasminogen activator (tPA) binding to the carboxyl-terminal lysine residue of the S100A10 subunit induces activation of ILK in a CD11b-dependent manner. Both tPA-mediated ILK activation and plasmin (via an unidentified receptor) promote NF-κB nuclear translocation where it induces production of proinflammatory mediators (TNFα, interleukins, *etc*.). (**b**) Progastrin binds to monomeric or dimeric annexin A2, but not AIIt, and induces clathrin-mediated endocytosis. Internalization of progastrin activates MAPKs, β-catenin, and nuclear translocation of NF-κB.

The annexin A2-S100A10 heterotetramer has been found to play a critical role in inflammation by mediating production of proinflammatory signals in response to various stimuli and also by promoting plasmin-dependent migration and infiltration of macrophages. Song *et al.*[220] demonstrated that lentiviral mediated RNA interference against S100A10 impairs LPS-induced production of the inflammatory cytokines TNFα, IL-1β and IL-10 as well as reduces activation of MAPKs and NF-κB in human chondrocytes. Interestingly, Lin *et al.*[221] demonstrated that tPA can induce NF-κB activity independently of its protease activity. Addition of tPA to macrophage cells promoted the aggregation of annexin A2 with integrin CD11b, consequently activating the integrin-linked kinase (ILK) pathway (Figure 4a) that promotes NF-κB activity by causing the phosphorylation and degradation of its inhibitor IκB. They further demonstrated that tPA induces NF-κB activity through binding to AIIt and not its other receptor, LDL receptor-related protein 1 (LRP-1). Additionally endogenously produced or exogenously added plasmin induces activation of monocytes and macrophages in a annexin A2 or S100A10 dependent manner via STAT3, NF-κB, ERK1 and 2 and p38 activation and induction TNF-α and IL-6 [222,223]. The annexin A2-S100A10 heterotetramer was shown to be essential in mediating this inflammatory response as knock-down of either S100A10 or annexin A2 impaired the plasmin-induced production of TNFα and IL-6 from macrophages [223]. The Hajjar laboratory found that plasmin induces Toll-like receptor 4 (TLR4)-dependent protein kinase C (PKC) activation in endothelial cells [224]. It has been suggested that plasmin induces TLR4-dependent signaling by proteolytically cleaving annexin A2 and causing its release from the annexin A2-S100A10 heterotetramer. The release of annexin A2 may be involved in the stimulation of TLR4 since addition of plasmin to endothelial cells induced protein-protein interactions between TLR4 and annexin A2; however, the mechanism of how plasmin stimulates inflammatory pathways is not entirely understood. Furthermore, plasmin-treated cells induced down-regulation of S100A10 protein level and reduced the surface level of annexin A2 without altering its cytoplasmic expression (Figure 4a). This indicates the existence of a negative feedback loop whereby plasmin induces the disassociation of the annexin A2-S100A10 heterotetramer, thus preventing further plasmin generation as well as production of proinflammatory signals [224]. Annexin A2 is known to act as a receptor of progastrin, which can stimulate proliferation via activation of NF-κB, β-catenin, and MAPKs [225]. The level of phospho-NF-κB(p65)^Ser276^ and β-catenin were down-regulation in colon crypts of annexin A2-null mice and in progastrin-expressing HEK-293 cells with annexin A2 knocked-down using siRNA [226]. Additionally, the down-regulation of annexin A2 in the rat intestinal cell line IEC-18 prevented the co-localization of progastrin and annexin A2 on the membrane and intracellularly, suggesting that annexin A2 is required for the binding and internalization of progastrin potentially via clathrin [227]. As discussed earlier, annexin A2, but not the AIIt, was shown to interact with the μ2 subunit of the clathrin assembly protein complex AP2 [124]. Treatment of cells with chlorpromazine (an inhibitor of clathrin-coated pit formation) impaired endocytosis of progastrin, indicating that annexin A2 mediates the endocytosis of progastrin in a clathrin-mediated manner (Figure 4b). The exact mechanism involving annexin A2 in clathrin-mediated endocytosis of progastrin to intercellular compartments is not entirely understood [227]. Together, these studies demonstrate that the AIIt has multiple roles in regulating the induction of inflammatory pathways and cytokine production by modulating the function of various membrane-associated proteins.

The annexin A2-S100A10 heterotetramer itself has also been shown to activate inflammatory pathways as a secreted product. The Feldman laboratory demonstrated that exogenous addition of the soluble annexin A2-S100A10 heterotetramer activates macrophages and promotes release of inflammatory cytokines via the MAPK and NF-κB pathways [198]. They further revealed that TLR4 is essential for annexin A2-S100A10 heterotetramer-mediated inflammatory signaling in murine and human macrophages [228]. Osteoclasts secrete AIIt, which acts in a paracrine/autocrine manner to stimulate differentiation of osteoclasts primarily via induction of GM-CSF and RANKL by activated T-cells and bone marrow stromal cells [229,230].

Plasmin mediated activation of MMP-9 has been shown to be a critical mediator of macrophage infiltration at inflammatory sites [176]. Our laboratory has shown using thioglycollate mediated peritonitis model that macrophage migration to the peritoneal cavity was significantly reduced in S100A10-null mice. Similarly *in vitro* evaluation of macrophage invasion using matrigel plugs demonstrated that S100A10-deficient macrophages showed significant reduction in invasion primarily by reduced plasmin generation and consequent MMP-9 activation suggesting a direct role of S100A10 in plasmin generation and inflammation [154]. Interestingly, we observed a decrease in annexin A2 protein levels in the S100A10-null macrophages which can be potentially attributed to changes in subcellular distribution of annexin A2. The direct role of annexin A2 in inflammation, can be assessed by immune and stress challenge of annexin A2-null mice. Unfortunately as discussed before, loss of annexin A2 in these mice also results in concomitant loss of S100A10, making interpretations of these experiments difficult.

### 9.12. Annexin A2 in Anti-Phospholipid Syndrome

Anti-phospholipid syndrome (APS) is an auto-immune disorder, where the immune system generates antibodies against phospholipid binding proteins. This disorder is characterized by increased risk for primary arterial and venous thrombosis, pregnancy miscarriages and other complications. Autoantibodies against the antigen β2 glycoprotein 1(β2-GPI), a member of the complement control protein is the primary perpetrator of APS. The plasma glycoprotein β2-GPI binds to phospholipids with lysine residues, and its phospholipid binding site is located at the carboxyl-terminal end in the [231,232] fifth of its five domains. This phospholipid binding property plays an important role in APS. Thrombosis is mediated by the autoantibodies that react to β2-GPI. The autoantibodies activate expression of endothelial cell adhesion molecules, secretion of inflammatory cytokines and increase pro-coagulant activity. The anti-β2-GPI complexes may also potentially trigger signaling events in leukocytes, endothelial cells, platelets leading to the expression pro-thrombocytic proteins. Annexin A2 was first identified as a high affinity binding partner for β2-GPI by affinity purification strategy/technology in Human Umbilical Vein Endothelial Cells (HUVEC) [233]. It was further observed that anti-β2-GPI antibodies activate endothelial cells through cross-linking annexin A2 bound β2-GPI [233,234]. Moreover it was observed that annexin A2 specific monoclonal antibodies which blocked β2-GPI binding to endothelial cells induced signaling pathways that present with a pro-adhesive and pro-coagulant phenotype. Consistently anti-annexin A2 specific antibodies are also observed in patients with APS which induced endothelial cell activation [235]. The detailed mechanism by which annexin A2 and β2-GPI induces signaling has not been clearly understood, primarily because annexin A2 is not a transmembrane protein. It is likely that some other adaptor protein which binds to annexin A2 is involved in mediating the signal leading to endothelial cell activation. It has been speculated that β2-GPI and TLR4 interact either directly or indirectly via annexin A2, thus mediating TLR4 signalling and NF-ΚB activation leading to the adhesive and coagulant phenotype [236]. TLR4 was purified from endothelial cells using annexin A2 immobilized columns [236]. Moreover β2-GPI and TLR4 interactions have been demonstrated in monocytes in which annexin A2 is richly expressed. Finally a recent investigation has demonstrated the existence of a signaling complex on the surface of endothelial cells consisting of annexin A2, TLR4, calreticulin, and nucleolin which mediates NF-κB dependent activation of endothelial cells by anti-β2-GPI antibodies [237]. But future studies are required to determine the precise nature of these interactions and specific role of annexin A2 in this signaling complex.

## 10. Concluding Comments

**Figure 5:**
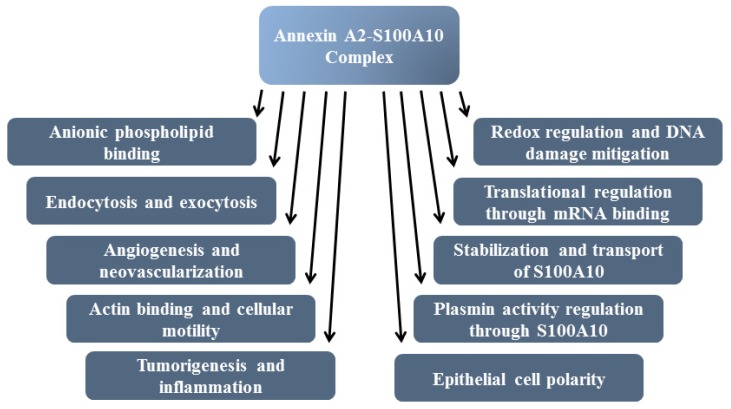
Functions of annexin A2-S100A10 complex. The schematic diagram summarizes the functions of annexin A2 discussed in the review.

Despite reports suggesting the key role of annexin A2 in a large number of important physiological processes ranging from exocytosis, endocytosis and the organization of lipid raft signaling domains, to ion channel transport to the plasma membrane, it is astounding that the annexin A2-null mouse shows no overt phenotype. This observation coupled with the demonstration that annexin A2 depletion or knockout causes changes in the cellular levels of other proteins such as S100A10 and fascin as well as effects the activity of over 60 genes strongly implies that a rigorous re-examination of the previous studies of the putative physiological function of annexin A2 is warranted. The validity of this suggestion is exemplified by the initial reports of the presence of fibrin in the tissues of the annexin A2-null mouse and followed by the conclusion that annexin A2 was a key regulator of plasmin generation and fibrinolysis. With the observation of dramatically diminished S100A10 levels in the annexin A2-null mouse and the recent report that S100A10-null mouse has pronounced deficits in fibrinolysis including fibrin deposition in the tissues, it is now apparent that annexin A2 does not play a direct role in fibrinolysis. Rather, annexin A2 stabilizes S100A10 protein levels and facilitates translocation of S100A10 to the cell surface where S100A10 binds tPA and plasminogen and regulates plasmin generation. Thus, the multitude of functions suggested for annexin A2 (Figure 5) will await further study and verification. However it is apparent that many of the functions of annexin A2 require the phospholipid-binding property of the protein. The identification of an important role of annexin A2 in redox regulation supported by observations in the annexin A2-null mouse and by *in vitro* data, suggests that redox regulation is a hitherto unexplored and exciting function of annexin A2. Elucidation of the full significance of this function will require further studies.

## Figures and Tables

**Table 1:** Characteristics of annexin A2 deregulation in cancer and the potential mechanisms. The table summarizes the effects of annexin A2 deregulation in multiple cancers as discussed in the review.

Cancer	Characteristics/Mechanisms	References
Breast cancer	-over-expression in invasive breast cancer and DCIS; absent in normal breast epithelium; predictor of response to neoadjuvant chemotherapy; tPA-mediated invasiveness and angiogenesis (through S100A10); metastatic cell proliferation (interaction with TNC); resistance to anthracyclines and taxanes.	[20,100,182–184]
PDAC	-over-expression in invasive lesions and PDAC; PDAC invasiveness and metastasis (probably through S100A10); activation of EMT; interaction with TNC in advanced pancreatic cancers; increased recurrence in post-operative patients pre-treated with gemcitabine; correlation with patient survival.	[83,87,185,190]
RCC	-over-expression in RCC patients (along with S100A10); low expression in normal renal tubules; AIIt as a potential diagnostic marker; -decreased metastasis-free survival of annexin A2-positive patients.	[191,192]
CRC	-correlation with increased tumor size, advanced histology and pTNM; plasmin-mediated invasiveness (through S100A10); correlation with TNC expression.	[193–195]
HCC	-over-expression in HCC tissues; activation of pro-inflammatory responses (through NF-κB); indicator of histological grade and improved reliability in diagnosis in HCC patients.	[197–200,202]
APL	-Activation of fibrinolysis (through S100A10); -ATRA and ATO treatments alleviate APL-associated bleeding by inhibiting annexin A2 and S100A10.	[204,205,207,208]
Prostate cancer	-annexin A2 and S100A10 expression lost especially in androgen-independent prostate cancer; prostate cancer cell migration; -annexin A2 down-regulation by DNA hypermethylation; **Conflicting results:** annexin A2 expression promotes cell proliferation, bone metastasis and tumor relapse	[84,168,210–212]
Head and neck cancers	ESCC -decreased expression in ESCC tissues; correlation with less differentiated ESCC tumors.	[210,214]
	NPC -reduced expression in NPC patients and cell lines; -implication with higher incidence of lymph node metastasis.	[215]
	HNSCC -down-regulation of annexin A2 in HNSCC patients; correlation with less differentiated tumors and lymph node metastasis; **Conflicting results:** annexin A2 down-regulation present in metastatic tumors and not primary tumors	[215–217]
